# RETRACTION: Effect of Humanised Care on the Surgical Site Wound Infection After Caesarean: A Meta‐Analysis

**DOI:** 10.1111/iwj.70278

**Published:** 2025-03-03

## Abstract

**RETRACTION:** WangX.‐Y. , WangX.‐Q. , TianZ.‐H. , LiW. , HeN. , and ZhangG.‐Q. , “Effect of Humanised Care on the Surgical Site Wound Infection After Caesarean: A Meta‐Analysis,” International Wound Journal 21, no. 4 (2024): e14547, 10.1111/iwj.14547.38098219 PMC10961047

The above article, published online on 14 December 2023, in Wiley Online Library (http://onlinelibrary.wiley.com/), has been retracted by agreement between the journal Editor in Chief, Professor Keith Harding; and John Wiley & Sons Ltd. Following an investigation by the publisher, all parties have concluded that this article was accepted solely on the basis of a compromised peer review process. The editors have therefore decided to retract the article. The authors did not respond to our notice regarding the retraction.

**RETRACTION:** X.‐Y. Wang , X.‐Q. Wang , Z.‐H. Tian , W. Li , N. He , and G.‐Q. Zhang , “Effect of Humanised Care on the Surgical Site Wound Infection After Caesarean: A Meta‐Analysis,” International Wound Journal 21, no. 4 (2024): e14547, 10.1111/iwj.14547.38098219 PMC10961047

The above article, published online on 14 December 2023, in Wiley Online Library (http://onlinelibrary.wiley.com/), has been retracted by agreement between the journal Editor in Chief, Professor Keith Harding; and John Wiley & Sons Ltd. Following an investigation by the publisher, all parties have concluded that this article was accepted solely on the basis of a compromised peer review process. The editors have therefore decided to retract the article. The authors did not respond to our notice regarding the retraction.

